# Adiponectin retards the progression of diabetic nephropathy in db/db mice by counteracting angiotensin II

**DOI:** 10.1002/phy2.230

**Published:** 2014-02-10

**Authors:** Xiaohua Guo, Guangyu Zhou, Meizi Guo, Alfred K Cheung, Yufeng Huang, Srinivasan Beddhu

**Affiliations:** 1 Division of Nephrology and Hypertension, Department of Internal Medicine, University of Utah School of Medicine, Salt Lake City, Utah; 2 Division of Nephrology, Department of Internal Medicine, Shengjing Hospital, China Medical University, Shenyang, 110004, China; 3 Medical Care Center, Veterans Affairs Salt Lake City Health Care System, Salt Lake City, Utah

**Keywords:** Adiponectin, diabetes, renal fibrosis, renin–angiotensin system

## Abstract

Adiponectin is a multifunctional adipokine with insulin‐sensitizing, anti‐inflammatory, and vasoprotective properties. Epidemiology studies have, however, shown that high levels of serum adiponectin are associated with kidney disease progression. We, therefore, examined the effect of adiponectin administration on the progression of glomerulosclerosis in the obese diabetic (db/db) mouse, a model of type II diabetes. Recombinant human adiponectin was administered intraperitoneally at a dose of 30 or 150 *μ*g per day from weeks 18 to 20. Rosiglitazone administered by gavage at 20 mg/kg body weight (BW) daily served as a therapeutic control. Untreated uninephrectomized db/db mice developed progressive albuminuria and glomerular matrix expansion, associated with increased expression of transforming growth factor beta 1 (TGF*β*1), plasminogen activator inhibitor type 1 (PAI‐1), collagen I (Col I), and fibronectin (FN). Treatment with adiponectin at either dose reduced the increases in albuminuria and markers of renal fibrosis seen in db/db mice, without affecting BW and blood glucose. Renal expressions of tumor necrosis factor‐*α* (TNF‐*α*) and monocyte‐chemoattractant protein‐1 (MCP‐1) and urinary TNF‐*α* levels, the markers of renal inflammation, were increased in diabetic mice, whereas adiponectin treatment significantly reduced the levels of these markers. Furthermore, adiponectin obliterated the stimulatory effects of angiotensin II (Ang II), but not the total effect of TGF*β*1, on the mRNA expression of PAI‐1, Col I, and FN by cultured glomerular mesangial cells. These observations suggest that adiponectin treatment reduces glomerulosclerosis resulting from type II diabetes probably through its anti‐inflammatory and angiotensin–antagonistic effects. Thus, adiponectin has therapeutic implications in the prevention of progression of diabetic nephropathy.

## Abstract (precis)

Adiponectin treatment reduces diabetic glomerulosclerosis probably through its anti‐inflammatory and renin–angiotensin‐system suppression properties. Importantly, unlike the proliferator‐activated receptor gamma‐(PPAR‐*γ*) ligands, the administration of adiponectin does not result in volume overload.

## Introduction

Diabetes is the leading cause of end‐stage renal disease (ESRD) and almost half of the incident ESRD patients in the United States have diabetes (Molitch et al. 2004). Despite the use of angiotensin‐converting enzymes inhibitors or angiotensin receptor blockers (Brenner et al. 2001; Parving et al. 2001), diabetic nephropathy progression is common. Hence, it is important to identify other potential interventions that might retard the progression of diabetic nephropathy.

Peroxisome proliferator‐activated receptor gamma‐(PPAR‐*γ*) agonists have shown promise in diabetic nephropathy (Bruno et al. 2005; Okada et al. 2006; Gross and Pistrosch 2007; Zheng and Guan 2007; Ko et al. 2008), however, these drugs are limited by potential side effects including volume overload, myocardial infarction (Nissen and Wolski 2007), and risk of bladder cancer (Inamoto et al. 2009).

PPAR*γ* agonists, such as rosiglitazone and pioglitazone, upregulate and increase the production of adiponectin by adipocytes (Yu et al. 2002). Adiponectin in turn increases insulin sensitivity (Kubota et al. 2002; McClain et al. 2005; Yamauchi et al. 2001). Further, experimental data suggest that adiponectin has direct effects on the kidney through binding to two receptors, adiponectin receptors 1 and 2. For example, increased urinary and glomerular markers of oxidative stress, podocyte foot process effacement, and albuminuria were observed in adiponectin knockout mice, while adiponectin administration reversed these abnormalities (Sharma et al. 2008). In the 5/6 nephrectomized model, treatment of adiponectin knockout mice, adenovirus‐mediated delivery of adiponectin resulted in the amelioration of albuminuria, glomerular hypertrophy, and tubulointerstitial fibrosis (Ohashi et al. 2007). However, it is unknown whether adiponectin therapy will affect obesity‐associated diabetic nephropathy. Therefore, we examined in a uninephrectomized model of obese diabetic (db/db) mice whether the administration of adiponectin decreased kidney inflammation and fibrosis, compared to the effects of rosiglitazone.

## Material and Methods

### Reagents

Unless otherwise indicated, all materials and chemicals were obtained from Sigma‐Aldrich (St. Louis, MO).

### Expression and purification of recombinant human adiponectin

Human full‐length adiponectin cDNA purchased from GeneCopoeia Inc. (Rockville, MD) was cloned into pET28a plasmid (EMD Chemicals Inc., Philadelphia, PA) with a polyhistidine (6× His) in N‐terminal to facilitate purification. The cloned adiponectin plasmid was transferred into *Escherichia Coli* for protein expression after it was confirmed by DNA sequencing. The fusion protein was produced in the form of inclusion bodies. It was purified and refolded by the on‐column methods (Oganesyan et al. 2005). Briefly, the inclusion bodies were solubilized in 6 mol/L urea, and loaded to a Ni^2+^‐charged iminodiacetic acid‐Sepharose resin column (5 mL, HiTrap Chelating HP, GE Healthcare, Salt Lake City, UT). After sequential washing with buffer containing 0.1% Triton X‐100 and buffer containing 5 mmol/L *β*‐cyclodextrin, adiponectin was eluted with imidazole gradient. The elution fractions were collected and the identity of adiponectin was confirmed by sodium dodecyl sulfate‐polyacrylamide gel electrophoresis (SDS‐PAGE) and western blotting analysis with a polyclonal rabbit anti‐human adiponectin IgG. The resulted adiponectin fusion protein was further confirmed by mass‐spectroscopy sequencing. Preparations with detectable endotoxin levels following phenyl‐Sepharose chromatography were further purified by EndoTrap Red affinity column (Hyglos GmbH, Bernried, Germany).

### In vivo studies of therapeutic effect of adiponectin in diabetic nephropathy

#### Animals

Diabetic male db/db mice (BKS.Cg‐Dock7^m^ +/+ Lepr^db^/J homozygotes, 000642) and their lean nondiabetic male db/m littermates were purchased from the Jackson Laboratory (Bar Harbor, ME) and housed in accordance with the National Institutes of Health Guide for the Care and Use of Laboratory Animals. The animal experiments were approved by the Animal Care Committee of University of Utah. The db/db mice were determined to be diabetic by the vendor on the basis of appearance of obesity at 5 week of age and were hyperglycemic when tested after arrival in our laboratory at week 7. Mice were subjected to right nephrectomy under anesthesia at week 8 to hasten the development of diabetic nephropathy as described previously (Huang et al. 2008). Db/m mice received uninephrectomy at week 8 served as the operation control.

#### Experimental design

Uninephrectomized male db/m normal mice were randomly assigned and treated at 18 weeks of age as follows: untreated at (*n* = 10) as healthy controls, treated with low dose (30 *μ*g, i.p., twice daily) of adiponectin for 2 weeks (*n* = 10), treated with rosiglitazone (20 mg/kg body weight [BW] by gavage daily) for 2 weeks (*n* = 9). Uninephrectomized male db/db diabetic mice were assigned and treated at 18 weeks of age as follows: untreated as diseased controls (*n* = 8), treated with adiponectin at two different dosages (30 *μ*g or 150 *μ*g, i.p., twice daily) for 2 weeks (*n* = 9 with each dosage), and treated with rosiglitazone (20 mg/kg BW by daily gavage) for 2 weeks (*n* = 7) as therapeutic control.

The tail blood glucose level was determined every 2 weeks using a blood glucose meter (Glucometer Elite XL, Bayer Healthcare, Elkhart, IN). Twenty‐four‐hour urine samples for the measurement of albumin were collected from each mouse in individual metabolic cages before treatment and prior to sacrifice. Urine albumin was measured using the DC 2000+ microalbumin/creatinine reagent kit (Bayer HealthCare). As for the reproducibility of this assay, the coefficients of variance (CV) were less than 3% when the same sample was measured three times consecutively. Urine tumor necrosis factor‐*α* (TNF‐*α*) levels were measured using a commercial available enzyme‐linked immunosorbent assay (ELISA) kit (Ebioscience, San Diego, CA). The kidneys were perfused through the heart with 30 mL of cold phosphate‐buffered saline (PBS) and then excised. Renal cortex was harvested by dissection and fixed in 4% neutralized formalin for histologic examination. In addition, pieces of cortex were stored in liquid nitrogen for western blotting or treated with TRIzol Reagent (Gibco BRL, Gaithersburg, MD) for RNA isolation or 100 mmol/L NaCl and 20 mmol/L hydroxyethyl piperazineethanesulfonic (HEPES) to be sonicated for 30 sec, three times on ice, and centrifuged at a high speed for 15 min at 4°C. The supernatant from the sonication was then collected and stored at −80°C for fibronectin (FN) ELISA (Assaypro, St. Charles, MO) and protein measurement by a bicinchoninic acid (BCA) protein assay kit (PIERCE, Rockford, IL).

#### Histological analysis

Three‐micrometer sections of paraffin‐embedded tissues were stained with periodic acid Schiff (PAS). All microscopic examinations were performed in a blinded manner. Glomerular matrix expansion was evaluated in 20 glomeruli from each mouse. The images (400× magnification) of 20 random glomeruli per slide were captured using a Nikon D50 digital camera and the PAS‐positive area in each glomerulus was quantified using a computer‐assisted color image analysis system (Image J, National Institutes of Health, Bethesda, MD). The PAS‐positive material area in the mesangium was divided by the glomerular tuft area to obtain an index of mesangial matrix as described previously (Huang et al. 2008).

#### RNA preparation and real‐time RT‐polymerase chain reaction

Total RNA was extracted immediately after retrieval from renal cortical tissues using the TRIzol Reagent according to the manufacturer's instructions. Two micrograms of total RNA was reverse‐transcribed using the superscript III first‐strand synthesis system (Invitrogen, Carlsbad, CA). Real‐time reverse transcription‐polymerase chain reaction (RT‐PCR) was performed using a SYBR green dye I (Applied Biosystems, Foster City, CA) with the ABI 7900 Sequence Detection System (Applied Biosystems). cDNA was first denatured at 95°C for 15 min and then amplified through 40 amplification cycles each consisted of denaturation at 95°C for 15 sec and annealing/extension at 60°C for 60 sec, according to the manufacturer's protocol. Fluorescence signals were recorded in each cycle. Relative quantification of gene expression was carried out using the 2ΔΔCT method and analyzed with RQ‐manager 1.2. (ABI 7900 Sequence Detection System; Applied Biosystems). Samples were run in triplicate in separate tubes to permit quantification of the target gene normalized to *β*‐actin. Sequences of primers used are listed in Table 1. The specificity of the PCR products was confirmed on a 1.5% agarose gel by showing a specific single band with the expected size.

**Table 1.** Primers used for real‐time reverse transcriptase‐PCR.

Gene	Primer	Location (complementary to nucleotides)	Sequence 5′–3′
Mouse TGF‐R1 (NM_011577.1)	Forward Reverse	1446–1467 1719–1738	TGA GTG GCT GTC TTT TGA CG TCT CTG TGG AGC TGA AGC AA
Mouse PAI‐1 (NM_008871.1)	Forward Reverse	1122–1141 1313–1330	AGT CTT TCC GAC CAA GAG CA ATC ACT TGC CCC ATG AAG AG
Mouse fibronectin (FN) (NM_010233.1)	Forward Reverse	7790–7807 7998–8015	CCG TGG GAT GTT TGA GAC GGC AAA AGA AAG CAG AGG
Mouse a1 (I) Collagen (NM 007742.3)	Forward Reverse	3765–3784 4014–4033	CAC CCT CAA GAG CCT GAG TC GCT TCT TTT CCT TGG GGT TC
Mouse TNF‐*α* (NM_013693)	Forward Reverse	327–345 440–459	TCCCCAAAGGGATGAGAAG CACTTGGTGGTTTGCTACGA
Mouse MCP‐1 (NM_011333)	Forward Reverse	173–194 237–259	GCCCCACTCACCTGCTGCTACT CCTGCTGCTGGTGATCCTCTTGT
Mouse beta‐actin(*β*‐actin) (NM_009606.1)	Forward Reverse	857–876 1132–1151	GCT CTT TTC CAG CCT TCC TT TGA TCC ACA TCT GCT GGA AG

PCR, polymerase chain reaction.

#### Western blot analysis

Equal amounts of renal cortical tissue (15 mg) from each mouse of each group were homogenized in a lysis buffer (Cell Signaling Technology, Beverly, MA) with 1% NP40, 1 mmol/L phenylmethylsulfonyl fluoride, and 1 tablet/5 mL protease inhibitor mixture (Complete, Mini; Roche Diagnostics Corp., Indianapolis, IN). Protein sample from 7 to 10 mice of each group was pooled for further examination. For western blot analysis, protein samples (20 *μ*g each group, determined by the BCA protein assay, Pierce, Rockford, IL) were separated by 10% Tris‐glycine gel electrophoresis (Invitrogen) and transferred to the Immobilon‐P transfer membrane (Millipore Corp., Bedford, MA). FN was detected using the rabbit anti‐human FN IgG as the primary antibody and the horseradish peroxidase‐conjugated goat anti‐rabbit IgG (Santa Cruz Biotechnology, Inc., Santa Cruz, CA) as the secondary antibody. The immunostained band was visualized and quantified as described previously (Huang et al. 2008). Briefly, bound antibodies were detected by developing the blot in enhanced chemiluminescence (ECL)™ western blotting detection reagents (Amersham Pharmacia Biotech, Little Chalfont, Buckinghamshire, U.K.). Quantitation of the bands on autoradiograms was performed using a Bio‐Rad GS‐700 imaging densitometer (Bio‐Rad Laboratories, Inc., Hercules, CA). Changes in FN protein expression were determined by normalizing against the densitometric intensity of *β*‐actin (detected using mouse monoclonal anti‐*β*‐actin IgG) or *α*‐tubulin (detected using mouse monoclonal anti‐*α*‐tubulin, Cell Signaling Technology). For comparison, this ratio was set at unity for normal control samples and other lanes on the same gel were expressed as fold change over this value. Three blots were performed for each primary antibody.

### In vitro studies of the effect of adiponectin on angiotensin II‐induced or TGF*β*1‐induced mesangial cell overexpression of PAI‐1 and matrix proteins

#### Cell culture

Mouse glomerular mesangial cell (MC) line purchased from ATCC (Manassas, VA) was cultured in Roswell Park Memorial Institute (RPMI) 1640 medium supplemented with 20% fetal bovine serum (FBS) (Hyclone Laboratory, Logan, UT), 100 U/mL penicillin, and 100 *μ*g/mL streptomycin at 37°C in a 5% CO_2_ incubator. Subconfluent cells seeded on 6‐well plates were made quiescent in serum‐free RPMI 1640 medium for 24 h before experimental studies. Mouse MCs were treated with 10^−6^ mol/L angiotensin II (Ang II) or 5 ng/mL transforming growth factor beta 1 (TGF*β*1) (R&D Systems, Inc., Minneapolis, MN) with or without recombinant adiponectin at the dose of 15 *μ*g/mL or 50 *μ*g/mL for 24 h.

#### Cellular RNA isolation and real‐time RT‐PCR

Total cellular RNA was isolated immediately from cultured MCs using Trizol Reagent (Gibco BRL) according to the manufacturer's instructions. Two micrograms of total RNA were reverse‐transcribed using the superscript III first‐stand synthesis system for RT‐PCR kit. Real‐time RT‐PCR was performed using a SYBR green dye I as described above.

#### FN western blot analysis

After 24‐h culture with or without adiponectin, MCs were collected in radioimmunoprecipitation assay buffer with protease inhibitors. Proteins were separated by 8% Tris‐glycine gel electrophoresis (Invitrogen) and transferred to PVDF membrane (Millipore). The membrane was subsequently blotted with the rabbit anti‐human FN IgG as described above.

### Statistical analysis

All data are expressed as mean ± SD. Statistical analyses of differences among the groups were performed by one‐way analysis of variance (ANOVA) and subsequent Student–Newman–Keuls or Dunnett testing for multiple comparisons. Comparisons with *P *< 0.05 were considered significantly different. In study 1, the disease‐induced increase in a variable was defined as the mean value for the disease control group minus the mean value of the normal control group (100%). The percent reduction in disease severity in a treated group was calculated as follows:
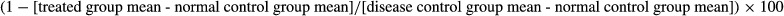


The urinary albumin excretion (UAE) values were log_10_‐transformed because of skewed distribution. As for a comparison in UAE, the log UAE was considered. In study 2, duplicate wells were analyzed for each experiment, and each experiment was performed independently a minimum of three times.

## Results

### Production of human recombinant adiponectin

**Figure 1.**
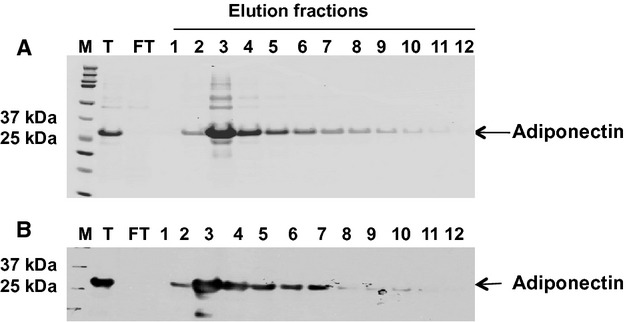
Elution of human recombinant adiponectin protein from a Ni^2+^‐charged iminodiacetic acid‐sepharose resin. The fractions were subjected to sodium dodecyl sulfate‐polyacrylamide gel electrophoresis (SDS‐PAGE) and Western blot analysis. (A) SDS‐PAGE of fractions from number 3 to number 10 developed with Coomassie blue shows a single band with an apparent molecular weight (Mr.) of 28 kDa. T, the total recombinant protein solubilized in 6 mol/L urea before applying onto the Ni2+‐charged column. FT, the flow through fraction collected when the recombinant protein was loaded on the column. There was no visible protein band in the flow through fraction, indicating that the recombinant protein binds to the column. (B) Western blot analysis of the same eluted fractions as in (A) using rabbit anti‐human adiponectin as the primary antibody, and horseradish peroxidase‐conjugated anti‐rabbit IgG as the secondary antibody. A single band that stains strongly for antiadiponectin appears in the fractions.

The recombinant human full‐length form of adiponectin made by the *E. Coli* expression system was applied to a modified Ni^2+^‐charged iminodiacetic acid column (5 mL) in 20 mmol/L imidazole buffer for purification. As shown in Figure 1A, the bound material was eluted by 250 mmol/L imidazole. The elution fractions were subjected to SDS‐PAGE and western blot assay. Bands were visualized by Coomassie blue staining (Fig. 1A). One specific protein band with the predicted size (~30 kDa) was seen in the elution fractions suggesting that the elution contained adiponectin, which was further confirmed by western blot assay as seen in Figure 1B. The resulted pure adiponectin fusion protein from the elution fraction 3 to 10 was pooled together and was subsequently dialyzed against PBS (pH 7.4). Its identity was further confirmed by mass‐spectroscopy sequencing (data not shown). Endotoxin levels for the final adiponectin preparations used in all experiments were below the Food and Drug Administration limit for parenteral drugs of five endotoxin units/kg, as assessed using the Chromogenic LAL Endotoxin Assay Kit (GenScript USA Inc., Piscataway, NJ).

### Therapeutic effects of adiponectin in diabetic nephropathy

#### Systemic effects

The baseline and final characteristics of the seven groups of mice are presented in Table 2. All mice survived until the time of sacrifice. The db/db mice remained hyperglycemic throughout the experimental period. No significant effect of adiponectin on glycemic control was seen in 2 weeks in db/db mice as the fasting plasma glucose concentration was not changed significantly by treatment with adiponectin (30 *μ*g or 150 *μ*g, i.p., twice daily for 2 weeks). At baseline, the BWs of db/db mice were significantly greater than those of db/m controls. The nondiabetic db/m mice gained ~1 g in BW from weeks 18 to 20. In contrast, the diabetic db/db mice lost 2–4 g in BW during the experimental period, except for those treated with rosiglitazone. Adiponectin treatment for 2 weeks had no effects on BWs in either db/m or db/db mice, when compared with the untreated db/m or db/db mice, respectively. In contrast, the rosiglitazone‐treated diabetic db/db mice had lower blood glucose levels and gained 0.73 g (*P* < 0.05 vs. untreated db/db mice). Presumably because of glycosuria, urine volumes were markedly increased in all db/db groups, compared with nondiabetic db/m mice. Urine volumes in db/db mice, except for the rosiglitazone‐treated group, increased by 2 mL per day by the end of experiment. In contrast, in rosiglitazone‐treated db/db mice, the urine volume significantly decreased by 6 mL per day by the end of experiment, indicating fluid retention, which may be the cause of the weight gain. The kidney weights (KW) were significantly greater in db/db control mice. However, the KW/BW ratio was not increased but significantly decreased in db/db mice due to the problem of obesity when compared with nondiabetic db/m control mice. The treatment with high dose of adiponectin or rosiglitazone significantly reduced the KWs in the db/db mice (*P* < 0.05). The ratio of KW/BW was not conclusive after treatment, particularly in the rosiglitazone‐treated db/db mice which may have uncorrect BW due to the potential fluid retention. These results indicate that diabetic db/db mice may develop kidney hypertrophy but the ratio of KW/BW is not a good marker of the organ hypertrophy in this model.

**Table 2.** Parameters of the experimental groups of mice.

	db/m (*n* = 10)	db/m + Ad‐30 (*n* = 10)	db/m + Ros (*n* = 9)	db/db (*n* = 8)	db/db + Ad‐30 (*n* = 9)	db/db + Adi‐150 (*n* = 9)	db/db + Ros (*n* = 7)
Plasma glucose, baseline, mg/dL	130 ± 22	125 ± 21	110 ± 15	507 ± 133^*^	489 ± 75^*^	445 ± 127^*^	515 ± 68^*^
Plasma glucose, end, mg/dL	103 ± 39	116 ± 23	129 ± 23	519 ± 147^*^	423 ± 114^*^	448 ± 152^*^	318 ± 161^*^^#^^§^
Δ body wt, g	0.78 ± 1.76	1.1 ± 2.19	1.0 ± 2.56	−3.66 ± 4.34^*^	−2.85 ± 1.98^*^	−1.93 ± 2.11^*^	0.729 ± 2.427^#^
Δ urine volume, mL	−0.18 ± 0.81	0.13 ± 0.92	−0.47 ± 1.12	2.25 ± 2.25^*^	1.19 ± 4.40	2.20 ± 6.08	−6.30 ± 4.52^*^^#^
UAE, baseline, log (*μ*g/day)	12.20 ± 3.39 1.07 ± 0.10	11.30 ± 3.26 1.03 ± 0.14	12.55 ± 3.24 1.08 ± 0.11	167.31 ± 138 2.09 ± 0.37^*^	180.47 ± 111 2.20 ± 0.21^*^	108.92 ± 96 1.92 ± 0.33^*^	153.06 ± 76 2.13 ± 0.22^*^
UAE, end, log (*μ*g/day)	11.02 ± 3.62 1.01 ± 0.16	12.55 ± 3.32 1.08 ± 0.11	10.76 ± 3.05 1.01 ± 0.12	487.89 ± 540 2.46 ± 0.47^*^^§^	171.12 ± 139 2.14 ± 0.27^*^	123.88 ± 78 2.00 ± 0.29^*^^#^	105.68 ± 67 1.95 ± 0.28^*^^#^
Kidney weight, g KW/BW, mg/g	0.264 ± 0.03 9.29 ± 0.67	0.263 ± 0.02 9.09 ± 0.089	0.263 ± 0.02 9.02 ± 0.92	0.317 ± 0.07^*^ 7.88 ± 2.22	0.281 ± 0.03 7.25 ± 0.74	0.262 ± 0.03^#^ 7.64 ± 1.98	0.255 ± 0.04^#^ 6.19 ± 1.67

Mice (nondiabetic db/m or diabetic db/db) were treated for 2 weeks continually with adiponectin (Ad), rosiglitazone (Ros) or PBS between 18 and 20 weeks of age. Unless specified, parameters were recorded at the end of the experimental period (20 weeks of age). wt, weight.*P < 0.05 versus db/m; ^§^*P* < 0.05 versus its own baseline; ^#^*P* < 0.05 versus db/db.

At the beginning of intervention (18 weeks of age), log10‐transformed UAE values in db/db mice were similar among the various groups of db/db mice and were all significantly higher than the various groups of db/m mice (Table 2). Albumin excretion increased between weeks 18 and 20 in untreated db/db mice (*P* < 0.05). This increase was completely prevented by high dose of adiponectin or rosiglitazone treatment (*P* < 0.05).

#### Exogenous adiponectin ameliorates fibrotic changes on diabetic kidneys

**Figure 2.**
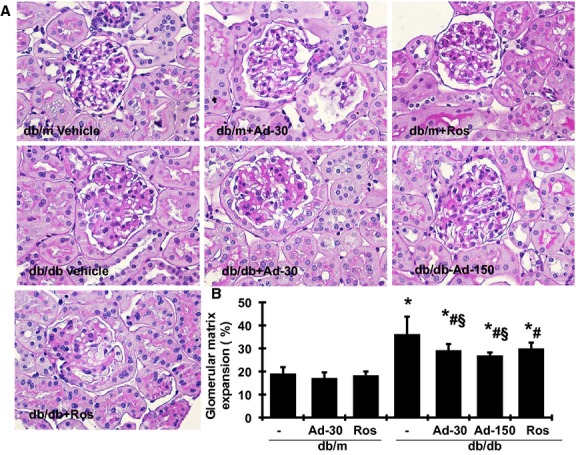
Effects of adiponectin and rosiglitazone on glomerular matrix protein accumulation. The histological sections stained with PAS are presented at 400 × magnification. (A) Representative photomicrographs of glomeruli at 20 weeks from db/m treated with vehicle, 30 *μ*g adiponectin (Ad) or rosiglitazone (Ros) and obese diabetic (db/db) mice treated with vehicle, 30 *μ*g or 150 *μ*g adiponectin or rosiglitazone. (B) Graphic representation of glomerular matrix score. *P < 0.05 versus db/m; ^#^*P <* 0.05 versus db/db; ^§^*P <* 0.05 vs. db/m treated with adiponectin.

Representative glomeruli stained with PAS are shown in Figure 2. Compared with db/m mice, untreated db/db mice at 20 weeks of age showed marked glomerular accumulation of extracellular matrix (ECM) appearing as PAS‐positive material and quantified by a computer‐assisted color image analyzer. Treatment with adiponectin substantially reduced glomerular mesangial matrix accumulation in db/db mice by 41.2% at low dose and 54.5% at high dose. Treatment with rosiglitazone in db/db mice resulted in a similar therapeutic effect on glomerular ECM accumulation when compared with the treatment with a low dose of adiponectin. Both adiponectin and rosiglitazone administration slightly reduced glomerular ECM accumulation in db/m, which was not statistically significant.

**Figure 3.**
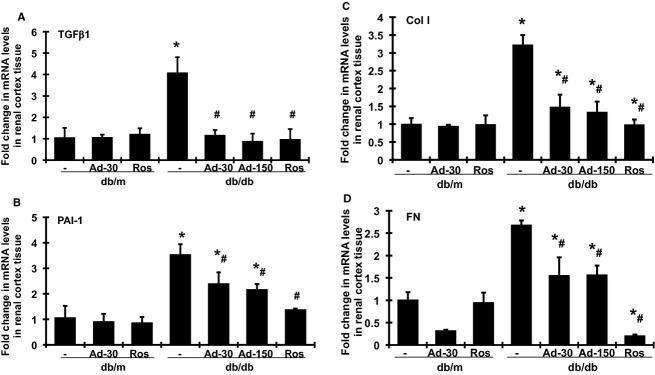
Effects of adiponectin and rosiglitazone on TGF‐*β*1, PAI‐1, and matrix protein mRNA expression in renal cortical tissues in diabetic db/db mice at week 20. Expression of mRNA was determined by real‐time RT‐PCR, correcting for the amplification of *β*‐actin. For comparison, this ratio was set at unity for untreated normal control (db/m) samples, and values of other groups were expressed as fold increase over this value. (A) Expression of TGF‐*β*1 mRNA. (B) Expression of PAI‐1 mRNA. (C) Expression of collagen I (Col I) mRNA. (D) Expression of FN mRNA. Ad‐30, 30 *μ*g adiponectin; Ad‐150, 150 *μ*g adiponectin was given at. Ros, rosiglitazone treated. *P < 0.05 versus db/m; ^#^*P <* 0.05 versus db/db.

Renal TGF‐*β*1, plasminogen activator inhibitor type 1 (PAI‐1), type I collagen (Col I), and FN mRNA expression was significantly increased in untreated db/db mice, compared with db/m mice at week 20 (Fig. 3, A–D). Adiponectin treatment effectively decreased the induction of TGF‐*β*1, PAI‐1, Col I, and FN in db/db mice in a dose‐dependent manner. Furthermore, TGF‐*β*1 expression in both adiponectin‐dose groups was reduced to the same levels as those seen in db/m mice.

Rosiglitazone treatment had no effect on renal mRNA expression of TGF*β*1, PAI‐1, and matrix protein in nondiabetic db/m mice. In contrast, rosiglitazone treatment in db/db mice completely abolished the increases in TGF*β*1, Col I, and FN mRNA expression and decreased the expression of PAI‐1 by 80%.

**Figure 4.**
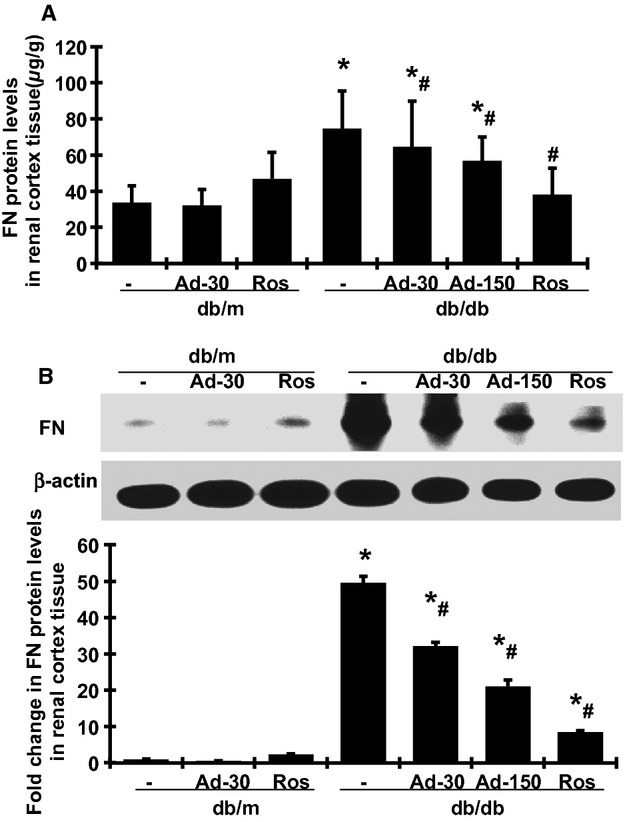
Effects of adiponectin and rosiglitazone on FN protein level in renal cortical tissues in diabetic db/db mice at week 20. (A) Total FN protein levels were measured by ELISA. (B) Representative western blots illustrating FN and *β*‐actin protein expression in each group. The lower graph summarizes the results of the band density measurements. Ad‐30, 30 *μ*g adiponectin; Ad‐150, 150 *μ*g adiponectin was given at. Ros, rosiglitazone treated. *P < 0.05 versus db/m; ^#^*P <* 0.05 versus db/db.

We further determined the protein levels of FN in renal cortex tissue, the major ECM protein that contributes to glomerular sclerosis. Consistent with the levels of FN mRNA expression, the levels of FN detected by ELISA (Fig. 4A) and western blot (Fig. 4B) in the renal cortex were markedly increased in db/db mice at week 20, compared with db/m mice (*P* *<* 0.05). Adiponectin administration significantly decreased the elevated protein levels of FN in db/db mice in a dose‐dependent manner. Rosiglitazone treatment had a greater effect on reducing the expression of FN protein in renal cortex of db/db mice, compared with the effect of adiponectin treatment.

### Exogenous adiponectin ameliorates renal inflammation in db/db mice

**Figure 5.**
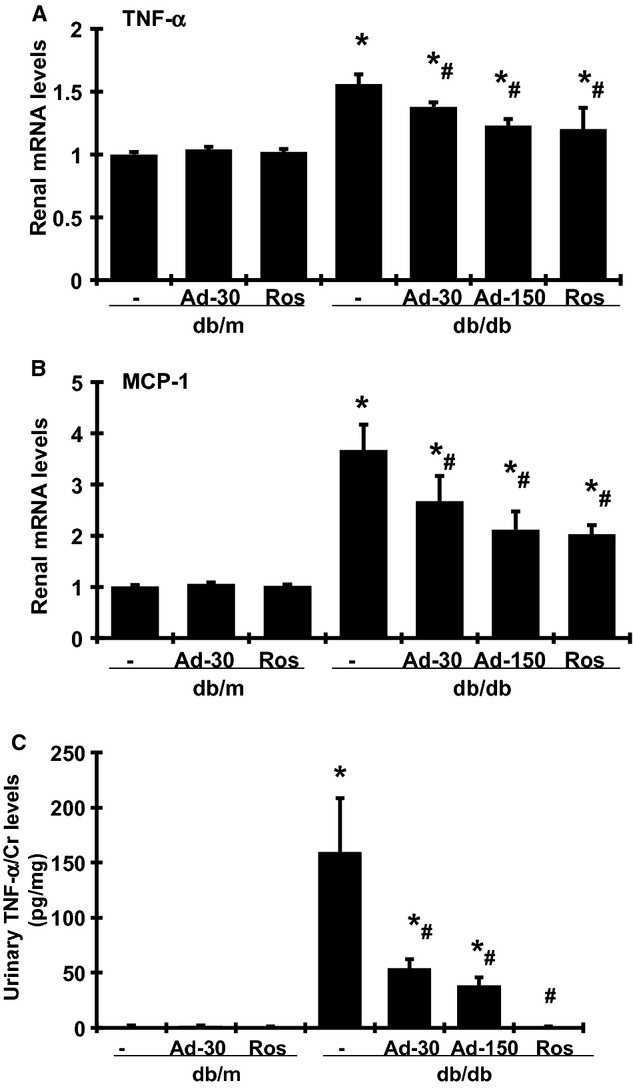
Effect of adiponectin and rosiglitazone on mRNA expression of renal TNF‐*α* (A) and MCP‐1 (B) in renal cortical tissues and urine TNF‐*α* levels (C) from nondiabetic (db/m) and diabetic db/db mice at week 20. Ad‐30, 30 *μ*g adiponectin; Ad‐150, 150 *μ*g adiponectin was given at. Ros, rosiglitazone treated. *P < 0.05 versus db/m; ^#^*P *< 0.05 versus db/db.

As renal inflammation contributes to the development of diabetic nephropathy (Wada and Makino 2013), we further investigated whether the renoprotective effect of adiponectin as described above is associated with a reduction in renal inflammation. As shown in Figure 5, renal TNF‐*α* and monocyte‐chemoattractant protein‐1 (MCP‐1) mRNA expression was significantly increased in the untreated db/db mice, compared with db/m mice. Urinary TNF‐*α* levels were also significantly increased in untreated diabetic mice (data not shown). As diabetic db/db mice had polyurine and lower levels of urinary creatinine at week 20 of age, urinary TNF‐*α* excretion that was corrected by urinary creatinine levels was markedly increased in the untreated db/db mice, compared with db/m mice. Adiponectin treatment significantly lowered the expression and production of TNF‐*α* and MCP‐1 in db/db mice in a dose‐dependent manner. Rosiglitazone treatment also reduced mRNA expression of these renal inflammation markers in db/db mice, similar to the treatment with the high dose of adiponectin. Urinary TNF‐*α* levels or urinary TNF‐*α* excretion was also significantly decreased in rosiglitazone‐treated db/db mice. As mentioned above, in rosiglitazone‐treated db/db mice, the urine volume significantly decreased and urinary creatinine increased compared with db/db mice, which may cause the levels of urinary TNF‐*α* further lower than those in adiponectin‐treated db/db mice.

### Effect of adiponectin on Ang II‐induced or TGF*β*1‐induced overexpression of PAI‐1 and matrix proteins in cultured MCs

**Figure 6.**
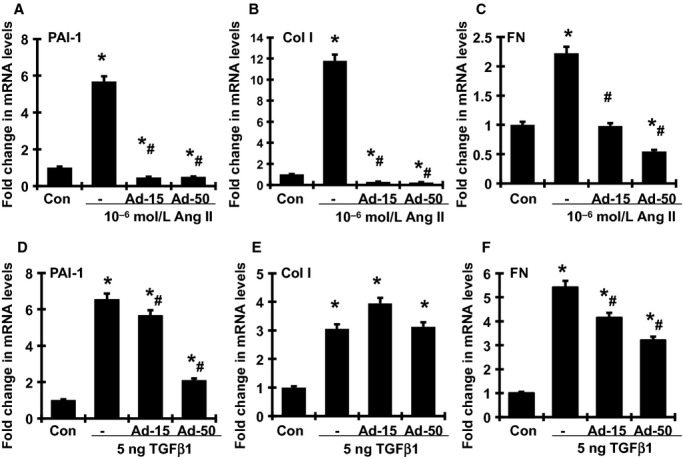
Dose‐dependent effect of adiponectin on Ang II‐induced (A–C) and TGF*β*1‐induced (D–F) PAI‐1, collagen I (Col I) and FN mRNA overexpression by mouse MCs detected by real‐time RT‐PCR. Ad‐15, 15 *μ*g/mL adiponectin; Ad‐50, 50 *μ*g/mL adiponectin. *P < 0.05 versus control cells (Con); ^#^*P <* 0.05 versus the Ang II alone or TGF*β*1 alone.

Administration of 10^−6^ mol/L Ang II or 5 ng/mL TGF*β*1 for 24 h significantly induced the mRNA expression of PAI‐1, Col I and FN in cultured mouse MCs when compared with medium‐alone control (Fig. 6A–F). Adiponectin treatment at doses of 15 *μ*g/mL or 50 *μ*g/mL blunted the increases in PAI‐1, Col I, and FN induced by Ang II in a dose‐dependent manner. In contrast, only high‐dose, but not low‐dose, adiponectin was effective in reducing the TGF*β*1‐induced Col I mRNA expression. The induction of PAI‐1 and FN by TGF*β*1 was significantly reduced, but not completely prevented, by pretreatment with adiponectin and this effect was dependent on the dose of adiponectin added (Fig. 6D and F).

**Figure 7.**
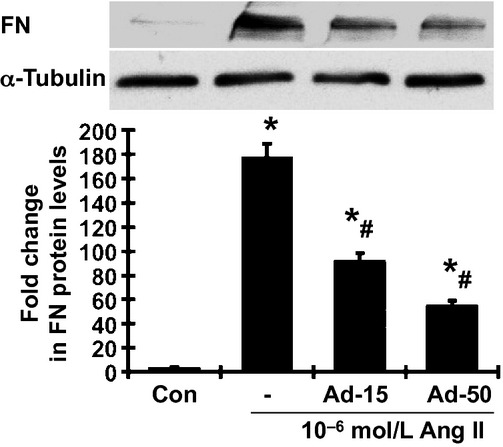
Effect of adiponectin on ANG II‐induced FN protein expression detected by western blot assay. Representative western blots illustrating FN and *α*‐tubulin protein expression. The lower graph summarizes the results of the band density measurements. Ad‐15, 15 *μ*g/mL adiponectin; Ad‐50, 50 *μ*g/mL adiponectin. *P < 0.05 versus control cells (Con); ^#^*P <* 0.05 versus the Ang II alone.

As shown in Figure 7, 10^−6^ mol/L Ang II markedly increased FN protein production in MCs, compared with medium alone. Adiponectin also significantly reduced the Ang II‐induced production of FN protein in a dose‐dependent manner.

## Discussion

Adiponectin, a 30‐kDa protein is secreted primarily by adipocytes and circulates as a multimer. It has been shown that adiponectin is anti‐inflammatory and reduces insulin resistance (Yamauchi et al. 2001; Kubota et al. 2002; McClain et al. 2005). Interestingly, although adiponectin is produced by the adipocyte, its secretion paradoxically decreases in obesity. This decrease in circulating adiponectin is thought to play an important role in inflammation and insulin resistance seen in obesity (Yamauchi et al. 2001; Kubota et al. 2002; McClain et al. 2005). Both experimental studies in animal models and epidemiological studies in humans (Choi et al. 2004) strongly suggest an insulin‐sensitizing property of adiponectin.

Nonetheless, the associations of adiponectin with kidney and cardiovascular outcomes in patients with chronic kidney disease or those on maintenance dialysis are controversial. In some cross‐sectional studies, serum adiponectin and albuminuria levels were inversely correlated (Tsioufis et al. 2005; Yano et al. 2007; Sharma et al. 2008), thus supporting a renoprotective role of adiponectin. In contrast, in another cross‐sectional study of Pima Indians with diabetes, higher serum adiponectin levels were associated with increased albuminuria and worse renal function (Looker et al. 2004). In the modification of diet in renal disease (MDRD) cohort with a predominantly nondiabetic population, higher baseline serum adiponectin levels were associated with increased proteinuria, cardiovascular, and all‐cause mortality (Menon et al. 2006). In a different study, men with serum adiponectin levels above 4 *μ*g/mL had significantly faster kidney disease progression (Kollerits et al. 2007). Whether the increases in serum adiponectin levels under these circumstances actually reflect the attempts to limit renal inflammation, and are therefore the consequence instead of the cause of kidney disease, remain unclear.

Animal studies have supported a protective role of adiponectin on renal tissues. Knockout mice for adiponectin display exacerbation of albuminuria, glomerular hypertrophy, and tubulointerstitial fibrosis, which are reversed by adiponectin treatment (Ohashi et al. 2007; Sharma et al. 2008). Adiponectin treatment also attenuated the ischemia reperfusion‐induced kidney dysfunction, inflammation, and apoptotic responses in mice (Becker et al. 2005). Further, overexpression of adiponectin using adenovirus‐mediated gene transfer alleviated the progression of proteinuria in streptozotocin (STZ)‐induced diabetic rats (Nakamaki et al. 2011).

We demonstrated herein the protective effect of adiponectin on renal tissue in a mouse model reflective of type II diabetic nephropathy in humans. The db/db mouse is a hyperinsulinemic model of genetic diabetes with obesity that develops abnormalities in renal morphology and function that parallel those in human nephropathy of type II diabetes. Our previous work on model development showed that performing uninephrectomy at 8 weeks of age greatly accelerated development and progression of diabetic nephropathy. Nonetheless, we acknowledge that uninephrectomy imposes a condition that does not usually exist in diabetes. In the present study, despite persistent hyperglycemia (Table 2), the increase in albuminuria was halted (Table 2) and the progression of glomerular mesangial matrix expansion was ameliorated (Fig. 2) by adiponectin administration. Consistent with previous observations in the db/db mouse, there were only slight pathological changes in the tubulointerstitial compartment (data not shown). The effect of adiponectin administration on renal tubulointerstitial injury was not able to be determined histologically. Rosiglitazone is known to induce adiponectin production and release by adipocytes, although it also confers other benefits unrelated to adiponectin (Sun et al. 2012). The administration of rosiglitazone for 2 weeks in the present study provided apparently greater renoprotective effects (Figs. 3, 4, and 5). Furthermore, administration of adiponectin or rosiglitazone in diabetic mice inhibited the renal expression of TGF*β*1, PAI‐1, Col I, and FN, proteins that are involved in the fibrotic pathway. Changes in the expression and production of these fibrotic markers may represent reduction in fibrosis in both glomerular and tubulointerstitial tissues. Collectively, these data on strategies that employ exogenous adiponectin or pharmacological induction of endogenous adiponectin release support an antifibrotic effect of this hormone in the kidney of db/db mice. Of note, the administration of adiponectin does not result in weight gain or decrease in urine volume. In contrast, the administration of rosiglitazone resulted in weight gain and a substantial decrease in urine output (Table 2), similar to the fluid retention seen in the administration of thiazolidinediones in human (Chaggar et al. 2009). In addition, rosiglitazone treatment but not adiponectin direct treatment, slightly reduced glucose levels in diabetic db/db mice in 2 weeks. These results suggest that the antifibrotic effect of adiponectin might be beyond its glucose‐lowering and insulin‐sensitizing effects that have been observed in humans.

As expected, we observed that two proinflammatory mediators, MCP‐1 and TNF‐*α*, were significantly upregulated in the renal cortical tissues of the db/db mice (Fig. 5). Previous reports have shown that urinary MCP‐1 and TNF‐*α* excretion increases in patients with diabetes, and the increment is even greater when albuminuria is severe (Liu et al. 2010; Navarro et al. 2006; Tashiro et al. 2002). These observations support the notion that the inflammatory pathways play a role in the progression of diabetic nephropathy. Our current study showed that adiponectin or rosiglitazone treatment markedly attenuated the elevated renal TNF‐*α* and MCP‐1 expression in the diabetic kidney (Fig. 5). Other groups have identified the anti‐inflammatory effect of adiponection in macrophages, monocytes, phagocytes, and tumor cells via inhibiting the activation of ERK1/2 and nuclear factor kappa beta‐dependent pathways, although some controversies remain. The anti‐inflammatory action of recombinant full‐length adiponectin that we observed in the present study may protect the kidney against injury in diabetes.

The data on cultured MCs revealed that the antifibrotic effect of adiponectin is mediated, at least in part, through antagonizing the effects of Ang II or TGF*β*, two key profibrotic factors in diabetic nephropathy. Although the mechanisms by which adiponectin modulates Ang II‐ or TGF*β*‐mediated profibrotic actions are unclear, increasing evidence suggests the signal link between Ang II and adiponectin. Ang II infusion decreased plasma concentrations of adiponectin and adipose tissue levels of adiponectin mRNA (Hattori et al. 2005). In contrast, treatment with Ang II blockers increased plasma adiponectin levels in both in humans and animals with metabolic syndrome or diabetes (Kurata et al. 2006; Fuke et al. 2010; Tian et al. 2010; Rinaldi et al. 2012). In addition to the inhibitory effect on adiponectin production by adipocytes, Ang II may also interact with adiponectin‐related intracellular signaling in the target cells. Adiponectin knockout mice exhibit the exacerbation of cardiac NADPH oxidase activation, a source of reactive oxygen species, and enhanced cardiac fibrosis in response to Ang II infusion (Fujita et al. 2008; Essick et al. 2011). Importantly, adiponection inhibited Ang II‐induced cell proliferation and cellular NF‐*κ*B activation in cultured neonatal rat ventricular myocytes (Essick et al. 2011). Excessive oxidative stress and inflammation have been implicated in cardiac hypertrophy and fibrosis. Taken together, these data suggest that the imbalance between adiponectin and Ang II could be a major determinant of cellular dysfunction in fibrotic diseases. Further, increasing adiponectin levels by targeting the action of Ang II may be a logical and promising approach for the treatment of cardiovascular and renal diseases associated with metabolic syndrome or diabetes. Further investigation of the pathophysiological interaction among adiponectin, Ang II, and TGF*β*, together with their interplay in signal transduction, is needed.

In summary, the findings in this present study both in vivo and in vitro indicate that adiponectin has antiprofibrotic functions. Adiponectin treatment reduces diabetic glomerulosclerosis probably through its anti‐inflammatory and renin–angiotensin‐system suppression properties. Importantly, unlike the PPAR‐*γ* ligands, the administration of adiponectin does not result in volume overload. Hence, exogenous adiponectin holds promise as a therapeutic agent in diabetic nephropathy and clinical interventional trials are needed to confirm or refute this hypothesis.

## Conflict of Interest

None declared.
